# Association of obesity status and metabolic syndrome with site-specific cancers: a population-based cohort study

**DOI:** 10.1038/s41416-020-1012-6

**Published:** 2020-07-30

**Authors:** Zhi Cao, Xiaomin Zheng, Hongxi Yang, Shu Li, Fusheng Xu, Xilin Yang, Yaogang Wang

**Affiliations:** 1 School of Public Health, Tianjin Medical University, Tianjin, China; 2 Department of Radiation Oncology, Anhui Provincial Hospital of Anhui Medical University, Hefei, China; 3 Department of Biostatistics, School of Public Health, Yale University, New Haven, USA

**Keywords:** Cancer epidemiology, Risk factors

## Abstract

**Background:**

Obesity and metabolic syndrome (MetS) appear in clusters and are both associated with an increased risk of cancer. However, it remains unknown whether obesity status with or without MetS increases the risk of site-specific cancers.

**Methods:**

We used data derived from 390,575 individuals (37–73 years old) from the UK Biobank who were enrolled from 2006–2016 with a median of 7.8 years of follow-up. Obesity was defined by BMI ≥ 30 kg/m^2^ and MetS was defined by the criteria of the Adult Treatment Panel-III (ATP-III). Cox proportional hazards models were used to investigate the associations of BMI and MetS with 22 cancers.

**Results:**

Metabolically healthy obesity (MHO) and metabolically unhealthy obesity (MUO) phenotypes represented 6.7% and 17.9% of the total analytic samples and 27.1% and 72.9% of the included subpopulation with obesity, respectively. Obesity was independently associated with higher risks of 10 of 22 cancers. Stratified by metabolic status, the MUO phenotype was consistently associated with 10 obesity-related cancers. In contrast, the MHO phenotype was only associated with increased risks of five cancers: endometrium, oesophagus, kidney, pancreas and postmenopausal breast cancers.

**Conclusion:**

Even in metabolically healthy individuals, obesity was associated with increased risks of five cancers, whereas we did not find that these individuals were associated with increased risks of several other obesity-related cancers.

## Background

The prevalence of overweight and obesity is increasing worldwide.^1^ Obesity predisposes individuals to a high risk of chronic non-communicable diseases (NCDs), such as cardiovascular disease and cancer.^2–5^ Indeed, obesity has resulted in a heavy disease burden and become a major public health issue. Obesity is often accompanied by metabolic syndrome (MetS), and both obesity and MetS contribute to an increased risk of NCDs.^6^ Obesity with MetS is known as metabolically unhealthy obesity (MUO). However, some individuals with obesity have few or no elevated metabolic risk factors (e.g. blood pressure, triglyceridemic, hyperglycaemia and low HDL cholesterol),^7^ suggesting that there may be a subset of individuals with obesity, who have metabolically healthy obesity (MHO).^8^ The proportion of those with MHO among the entire population with obesity ranges from 10–40%, depending on the population under study and the criteria used to define MetS.^9^ A previous study in the UK showed that 9.3% of participants were affected by obesity and that 42.5% of them were classified as the MHO phenotype.^10^ The classification of obesity into MUO and MHO phenotypes has been increasingly recognised and used by clinicians and public health practitioners in their clinical and public health practices, e.g. in determining clinical treatments for obesity.^11^

MHO individuals have less visceral fat mass, lower ectopic fat deposition, and more favourable inflammatory and hormonal profiles than their metabolically unhealthy counterparts.^11^ Thus, it is unclear whether obesity without MetS actually increases the risk of NCDs. Some studies have reported that the MHO phenotype does not increase the risk of cardiovascular disease or diabetes.^12^ Likewise, previous studies have found that individuals with the MHO phenotype were at a lower risk of colorectal cancer.^13,14^ However, there is also some evidence that postmenopausal women with the MHO phenotype may be at an increased risk for breast cancer.^15–17^ Although studies have repeatedly shown that obesity is associated with an increased risk of obesity-related cancers,^18,19^ it remains unknown whether the MHO phenotype predisposes individuals to high risks of all types of cancer or only site-specific cancers. Therefore, in the present study, we analysed data from a longitudinal UK Biobank cohort to evaluate potential associations of MetS and obesity phenotypes with the development of 22 types of cancer in the general UK population.

## Methods

### Study design and population

This was a prospective, population-based cohort study of participants enrolled in the UK Biobank. Between April 2006 and December 2010, the UK Biobank recruited 502,528 adults aged 40–69 years from the general population. Participants visited one of 22 assessment centres across England, Scotland and Wales, where they completed touchscreen and nurse-led questionnaires, had physical measurements taken and provided biological samples.^20^ Since recruitment at baseline between 2006 and 2010, participants were followed up for the incidence of cancer until 2016. In our present study, we excluded participants who had a diagnosis of cancer (other than nonmelanoma skin cancer, based on the International Classification of Diseases, 10th Revision [ICD-10] code C44) at baseline between 2006 and 2010 (*n* = 41,491), a body mass index (BMI) < 18.5 kg/m^2^ (*n* = 2357) or missing information on BMI or metabolic abnormalities (68,105), leaving data from 390,575 remaining participants to be included in the present study (Supplementary Fig. 1).

### Exposure and covariate assessments

We used standard operating protocols to measure four Adult Treatment Panel-III (ATP-III) components to define MetS, as follows:^21^ (1) elevated blood pressure (BP), defined as a systolic BP ≥ 130 and/or a diastolic BP ≥ 85 mmHg and/or the use of antihypertensive medication at baseline; (2) hypertriglyceridemia, defined as triglycerides ≥1.7 mmol/L (150 mg/dL) or current use of lipid-lowering medication at baseline; (3) low HDL cholesterol, defined as <1.0 mmol/L (40 mg/dL) for men and <1.3 mmol/L (50 mg/dL) for women; (4) hyperglycaemia, defined as fasting blood glucose ≥5.6 mmol/L or use of medications for diabetes at baseline (e.g. insulin or oral antidiabetic medications). Participants with two or more of the four criteria were considered to have MetS.^10,22,23^ Different criteria for the metabolically healthy classification were used in sensitivity analyses. Obesity was defined as a BMI ≥ 30 kg/m^2^, overweight was defined as 25 ≤ BMI < 30 kg/m^2^, and normal weight was defined as 18.5 ≤ BMI < 25 kg/m^2^. In the combined analysis of BMI and MetS, participants were divided into six phenotypes: metabolically healthy normal weight (MH-NW), metabolically unhealthy normal weight (MU-NW), metabolically healthy overweight (MH-OW), metabolically unhealthy overweight (MU-OW), MHO and MUO.

Waist circumference is also a diagnostic indicator of obesity, especially central obesity. We also conducted an analysis of the associations of combined central obesity and MetS (not including waist circumference) with 22 incident cancers. In the main analysis, waist circumference was not included as a criterion of MetS because of collinearity with BMI when we explored the joint effect of BMI and MetS on incident cancers.

The potential confounders of our analyses included socio-demographic, behavioural and lifestyle factors and health status and treatments related to cancer morbidities. Socio-demographic variables included age, sex, ethnicity, education attainment, employment and the Townsend deprivation index. The Townsend deprivation index was assigned as a continuous measure based on postcodes, which were derived from census data on housing, employment, social class and car availability, a higher index indicated more deprivation. Behavioural and lifestyle factors included smoking status and alcohol intake. Health status and treatments included a history of hormone replacement therapy (HRT), hysterectomy, menopause, diabetes and cardiovascular disease. Detailed information regarding how these variables were collected is provided in the Supplementary Methods.

### Outcome ascertainment

Participants were followed up for incident cancer until the end of the study (November 17, 2016) via the NHS Central Register, which provides information on cancer registrations and deaths. The end point for each participant included in these analyses was the first diagnosis of cancer, loss to follow-up or death, whichever event occurred first. We defined cancer using the ICD-10 (Supplementary Table 1). The outcomes assessed in this analysis consisted of 22 types of cancer with the highest incidences, as follows:^24^ oral, oesophagus, stomach, colorectal, liver, gallbladder, gallbladder, pancreas, lung, malignant melanoma, postmenopausal breast (breast cancer that occurred after menopause), cervix, endometrium, ovary, prostate, kidney, bladder, brain and thyroid cancers, and non-Hodgkin lymphoma, multiple myeloma and leukaemia.

### Statistical analyses

The characteristics of the study sample are presented as the mean + standard deviation (SD) or as a percentage when appropriate across BMI categories. Person-years were calculated from the date of recruitment to the onset of the 22 types of incident cancer or the censoring date, whichever event occurred first.

In the first set of analyses, Cox proportional hazard regression models with attained age as a timescale were utilised to control for the confounding effects of age in the examination of the relationship between BMI categories and the risks of 22 types of cancer. The proportional hazard assumption was checked by tests based on Schoenfeld residuals, and the results indicated that the assumptions were not violated. Hazard ratios (HRs) and 95% confidence intervals (95% CIs) were obtained by adjusting for sex, age, education attainment, employment, ethnicity, Townsend deprivation index, alcohol intake and smoking status. Models for cervix, ovary and endometrium cancers were also adjusted for HRT use, oral contraceptive use and menopause after excluding females with a history of hysterectomy (*N* = 13,743). Models for postmenopausal breast cancer were also adjusted for HRT and oral contraceptive use. We also evaluated the role of metabolic status in BMI–cancer associations by running all models with and without adjustment for metabolic status. The difference in HR was calculated as the percent change from preadjustment to postadjustment for metabolic status. We then ran a second set of analyses, stratified by metabolic status to allow us to compare the risks of incident cancers as a function of overweight and obese status. The normal-weight group was the reference group in these analyses. Missing and/or unknown values were assigned to a separate category when the variable was included as a covariate (missing proportions less than 1%).

We performed a series of analyses to assess the robustness of our findings. First, we repeated the main analysis after excluding the first 2 years of follow-ups to minimise the potential for reverse causality. Second, we included waist circumference as a criterion (elevated waist circumference was defined as a waist circumference ≥ 94 cm for men and a waist circumference ≥ 80 cm for women) and defined metabolic health as having less than three of five possible metabolic abnormalities (e.g. elevated blood pressure, hypertriglyceridemia, hyperglycaemia, low HDL cholesterol and elevated waist circumference).^7,25^ Third, we reanalysed the data after excluding subjects who had a history of cardiovascular disease (e.g. heart attack, angina and stroke) or diabetes at baseline, both of which may affect metabolic status. Finally, we used a multiple imputation approach to impute the missing values for nonsystematically missing covariables. Five imputed datasets were generated and estimates were combined using Rubin’s rules. All analyses were performed using STATA 15 statistical software (StataCorp) and R i386 3.4.3 (R Foundation for Statistical Computing). All *P-*values were two‐sided, and a *P* < 0.05 was considered statistically significant.

## Results

**Fig. 1:**
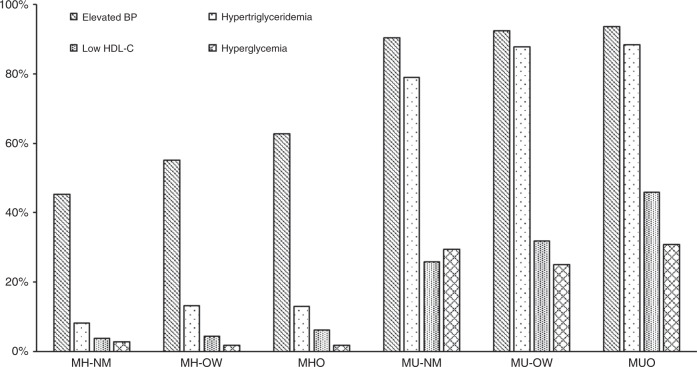
Distribution of MetS criteria by metabolic health and obesity status. MetS metabolic syndrome, MH-NW metabolically healthy normal weight, MH-OW metabolically healthy overweight, MHO metabolically healthy obesity, MU-NW metabolically unhealthy normal weight, MU-OW metabolically unhealthy overweight, MUO metabolically unhealthy obesity.

We ultimately included 390,575 participants who participated in the 2006–2010 UK study, among whom 36,638 (9.4%) had incident cancers. The median follow-up period was 7.8 years for all of the 22 types of cancer. Table 1 summarises the main characteristics of the participants by BMI and metabolic health categories at baseline. Among participants, 53% were women, and the median age at baseline was 56.3 years. The MHO and MUO phenotypes represented 6.7% (*n* = 26,094) and 17.9% (*n* = 70,079) of the total analytic sample and 27.1% and 72.9% of the population with obesity, respectively. Blood pressure, fasting glucose and triglycerides were higher in MUO individuals, while HDL cholesterol was higher in MHO individuals. Figure 1 shows the distribution for MetS by ATP-III criteria. Four components were more prevalent in those who were metabolically unhealthy than in those who were metabolically healthy. The most common MetS criterion was high blood pressure, followed by hypertriglyceridemia, low HDL-C, and hyperglycaemia. We also report information according to BMI categories, which can be found in Supplementary Table 2.

**Table 1:** Baseline characteristics of participants across BMI and metabolic status.

Characteristics	Total (390 575)	Metabolically healthy			Metabolically unhealthy		
		Normal weight	Overweight	Obesity	Normal weight	Overweight	Obesity
Sex							
Female	206,954 (53.0)	61,774 (68.3)	41,829 (53.8)	16,171 (62.0)	19,384 (53.3)	34,685 (38.6)	33,111 (47.2)
Male	183,621 (47.0)	28,736 (31.7)	35,857 (46.2)	9923 (38.0)	16,963 (46.7)	55,174 (61.4)	36,968 (52.8)
Age (mean, SD)	56.3 (8.1)	54.2 (8.1)	55.0 (8.3)	54.7 (7.9)	58.4 (7.7)	58.2 (7.8)	57.3 (7.8)
40–49 years	95,243 (24.4)	15,962 (44.0)	8086 (40.8)	1806 (42.3)	19,833 (21.9)	30,076 (20.4)	19,480 (21.2)
50–59 years	131,602 (33.7)	13,037 (36.0)	7223 (36.5)	1624 (38.0)	30,143 (33.3)	47,578 (32.2)	31,997 (34.8)
60–70 years	163,730 (41.9)	7267 (20.0)	4487 (22.7)	841 (19.7)	40,615 (44.8)	70,095 (47.4)	40,425 (44.0)
Ethnicity							
White	367,393 (94.1)	85,982 (95.0)	73,112 (94.1)	23,848 (93.4)	33,936 (93.4)	84,576 (94.1)	65,939 (94.1)
Mixed background	2339 (0.6)	641 (0.7)	508 (0.7)	223 (0.9)	184 (0.5)	409 (0.5)	374 (0.5)
South Asian	7834 (2.0)	1359 (1.5)	1216 (1.6)	350 (1.30)	1191 (3.3)	2394 (2.7)	1324 (1.9)
Black	6340 (1.6)	923 (1.0)	1634 (2.1)	1172 (4.5)	277 (0.8)	996 (1.1)	1338 (1.9)
Chinese	1231 (0.3)	504 (0.6)	149 (0.2)	14 (0.1)	274 (0.8)	239 (0.3)	51 (0.1)
Others	3599 (0.9)	748 (0.8)	700 (0.9)	340 (1.3)	328 (0.9)	820. (0.9)	663 (0.9)
Missing	1839 (0.5)	353 (0.4)	367 (0.5)	147 (0.6)	157 (0.4)	425 (0.5)	390 (0.6)
Townsend deprivation index							
1 (least deprived)	78,572 (20.1)	19,809 (21.9)	16,514 (21.3)	4522 (17.3)	7660 (21.1)	18,505 (20.6)	11,562 (16.5)
2	78,041 (20.0)	18,599 (20.5)	16,133 (20.8)	4788 (18.3)	7644 (21.0)	18,365 (20.4)	12,512 (17.9)
3	77,525 (19.8)	18,052 (19.9)	15,883 (20.4)	5077 (19.5)	7164 (19.7)	18,078 (20.1)	13,271 (18.9)
4	78,556 (20.1)	17,992 (19.9)	15,365 (19.8)	5470 (21.0)	7075 (19.5)	17,825 (19.8)	14,829 (21.2)
5 (most deprived)	77,404 (19.8)	15,955 (17.6)	13,689 (17.6)	6208 (23.8)	6761 (18.6)	1698 (18.9)	17,809 (25.4)
Missing	477 (0.1)	103 (0.1)	102 (0.1)	29 (0.1)	43 (0.1)	104 (0.1)	96 (0.1)
Employment							
Working	229,089 (58.7)	59,972 (66.3)	51,245 (66.0)	16,994 (65.1)	17,569 (48.3)	46,446 (51.7)	36,863 (52.6)
Retired	124,975 (32.0)	22,068 (24.4)	20,393 (26.3)	6287 (24.1)	15,427 (42.4)	36,132 (40.2)	24,668 (35.2)
Unemployed	29,487 (7.6)	6668 (7.3)	4648 (6.0)	2304 (8.8)	2657 (7.3)	5855 (6.5)	7355 (10.5)
Other	7024 (1.8)	1802 (2.0)	1400 (1.8)	509 (2.0)	694 (1.9)	1426 (1.6)	1193 (1.7)
Education attainment							
College degree	127,067 (32.5)	38,407 (42.4)	26,858 (34.6)	7261 (27.8)	12,188 (33.5)	26,035 (29.0)	16,318 (23.3)
A levels/AS levels	43,588 (11.2)	11,519 (12.7)	9100 (11.7)	2904 (11.1)	3871 (10.7)	9169 (10.2)	7025 (10.0)
O levels/GCESs	82,516 (21.1)	18,229 (20.1)	16,984 (21.9)	5999 (23.0)	7487 (20.6)	18,743 (20.9)	15,074 (21.5)
CSEs	21,469 (5.5)	4496 (5.0)	4651 (6.0)	1904 (7.3)	1579 (4.3)	4481 (5.0)	4358 (6.2)
NVQ or HND or HNC	25,833 (6.6)	4025 (4.5)	4914 (6.3)	1837 (7.0)	2206 (6.1)	7057 (7.8)	5794 (8.3)
Other Qualifications	20,014 (5.1)	3987 (4.4)	3708 (4.8)	1349 (5.2)	1997 (5.5)	4974 (5.5)	3999 (5.7)
None of the above	65,480 (16.8)	9131 (10.1)	10,660 (13.7)	4502 (17.3)	6550 (18.0)	18,224 (20.3)	16,413 (23.4)
Missing	4608 (1.2)	716 (0.8)	811 (1.0)	338 (1.3)	469 (1.3)	1176 (1.3)	1098 (1.6)
Smoking status							
Never	213,724 (54.7)	54,938 (60.7)	44,119 (56.8)	14,624 (56.0)	20,039 (55.1)	45,557 (50.7)	34,447 (49.2)
Previous	133,744 (34.2)	25,914 (28.6)	26,128 (33.6)	9070 (34.8)	11,065 (30.4)	33,559 (37.3)	28,008 (40.0)
Current	41,173 (10.5)	9341 (10.3)	7089 (9.1)	2260 (8.7)	5075 (14.0)	10,261 (11.4)	7147 (10.2)
Missing	19.34 (0.5)	317 (0.4)	350 (0.5)	140 (0.5)	168 (0.5)	482 (0.5)	477 (0.7)
Alcohol intake frequency							
Daily or almost daily	79,429 (20.3)	20,228 (22.3)	16,392 (21.1)	4032 (15.5)	8415 (23.2)	19,684 (21.9)	10,678 (15.2)
Three or four times a week	90,723 (23.2)	23,341 (25.8)	19,976 (25.7)	5399 (20.7)	7960 (21.9)	20,999 (23.4)	13,048 (18.6)
Once or twice a week	100,933 (25.8)	23,145 (25.6)	21,024 (27.1)	7236 (27.7)	8826 (24.3)	22,710 (25.3)	17,992 (25.7)
One to three times a month	43,480 (11.1)	9436 (10.4)	8131 (10.5)	3477 (13.3)	3636 (10.0)	9403 (10.5)	9397 (13.4)
Special occasions only	44,268 (11.3)	8327 (9.2)	7261 (9.3)	3683 (14.1)	4177 (11.5)	9630 (10.7)	11,190 (16.0)
Never	30,894 (7.9)	5877 (6.5)	4760 (6.1)	2197 (8.4)	3245 (8.9)	7240 (8.0)	7575 (10.8)
Missing	848 (0.2)	156 (0.2)	142 (0.2)	70 (0.3)	88 (0.2)	193 (0.2)	199 (0.3)
Systolic BP (mmHg; mean, SD)	138.1 (18.0)	129.7 (17.4)	134.0 (17.4)	136.4 (17.9)	142.7 (16.8)	144.0 (16.4)	143.8 (16.6)
Diastolic BP (mmHg; mean, SD)	82.5 (9.8)	77.8 (9.3)	81.2 (9.3)	84.3 (9.6)	82.5 (9.3)	84.9 (9.4)	86.5 (9.5)
HDL cholesterol (mmol/L; mean, SD)	1.44 (0.38)	1.69 (0.37)	1.54 (0.34)	1.48 (0.30)	1.44 (0.39)	1.29 (0.32)	1.20 (0.28)
Triglycerides (mmol/L; mean, SD)	1.75 (1.03)	1.12 (0.46)	1.30 (0.57)	1.36 (0.58)	1.92 (0.96)	2.28 (1.12)	2.42 (0.20)
Glucose (mmol/L; mean, SD)	5.12 (1.24)	4.79 (0.56)	4.83 (0.51)	4.86 (0.48)	5.33 (1.42)	5.32 (1.41)	5.61 (1.89)

Values are numbers (percentages) unless stated otherwise

*BMI* body mass index (calculated as weight in kilograms divided by height in meters squared), *BP* blood pressure, *CSE* Certificate of Secondary Education, *GCSE* General Certificate of Secondary Education, *HNC* Higher National Certificate, *HND* Higher National Diploma, *NVQ* National Vocational Qualification.

**Fig. 2:**
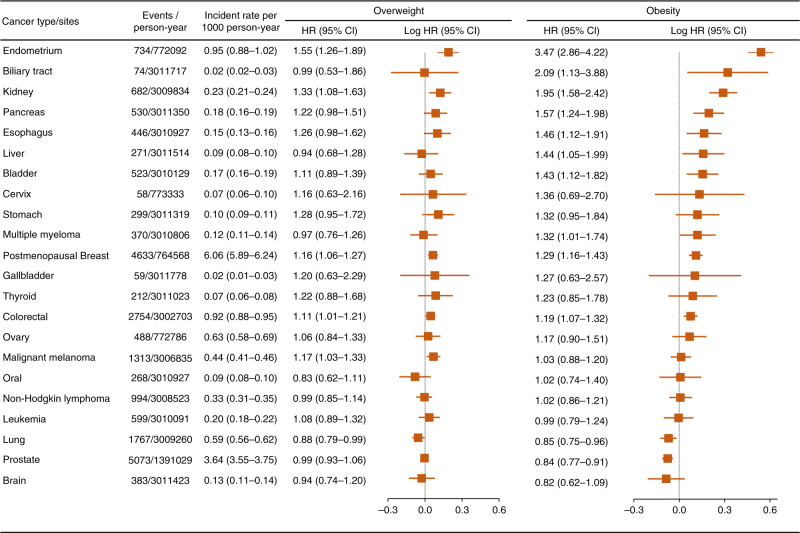
Incidence rate per 1000 person-year and multivariable HRs of cancers according to BMI categories. HRs of overweight and obesity were compared with normal weight. Multivariable models were adjusted for sex, age, ethnicity, Townsend deprivation index, qualification, employment status, alcohol intaking, smoking status. Models for cervix, ovary and endometrium cancers are additionally adjusted for HRT use, oral contraceptive use and menopause after excluded females with history of hysterectomy. Models for postmenopausal breast was additionally adjusted for HRT and oral contraceptive use.

BMI was independently associated with 12 of 22 cancers (Fig. 2). Compared with those of normal weight, individuals with obesity had positive associations for 10 cancers: endometrium, biliary tract, kidney pancreas, oesophagus, liver, bladder, postmenopausal breast and colorectal cancers and multiple myeloma. However, obesity was associated with a lower risk of lung cancer (HR = 0.85, 95% CI, 0.75–0.96) and prostate cancer (HR = 0.84, 95% CI, 0.77–0.91).

Adjusting for metabolic health attenuated the associations for endometrium, liver and kidney cancer (Table 2) and nullified the association for liver cancer; that is, associations for liver cancer were no longer statistically significant in metabolic health adjusted models, although the HRs were still consistent with a 22% higher risk of liver cancer. Associations for multiple myeloma were strengthened after adjustment for metabolic status. Otherwise, the effects of adjustment for metabolic health were modest, and 11 of 12 associations remained statistically significant after adjustment.

**Table 2:** Comparison of multivariable HRs for obesity vs normal-weight level of BMI by cancer type, without and with adjustment for metabolic status.

Cancer type/sites	Hazard ratio (95% CI)		Difference in HR, %
	Not metabolic status adjusted^a^	Metabolic status adjusted^b^	
Endometrium	3.47 (2.86–4.22)	2.94 (2.39–3.62)	15.3
Liver	1.44 (1.05–1.99)	1.22 (0.86–1.72)	15.3
Kidney	1.95 (1.58–2.42)	1.69 (1.35–2.13)	13.3
Bladder	1.43 (1.12–1.82)	1.32 (1.02–1.71)	7.7
Esophagus	1.46 (1.12–1.91)	1.35 (1.02–1.80)	7.5
Lung	0.85 (0.75–0.96)	0.79 (0.69–0.91)	7.1
Pancreas	1.57 (1.24–1.98)	1.46 (1.13–1.88)	7.0
Colorectal	1.19 (1.07–1.32)	1.13 (1.01–1.26)	5.0
Postmenopausal breast	1.29 (1.16–1.43)	1.26 (1.13–1.41)	2.3
Biliary tract	2.09 (1.13–3.88)	2.06 (1.07–3.97)	1.4
Prostate	0.84 (0.77–0.91)	0.85 (0.78–0.93)	−1.2
Multiple myeloma	1.32 (1.01–1.74)	1.40 (1.05–1.88)	−6.1

Models for cervix, ovary and endometrium cancers are additionally adjusted for HRT use, oral contraceptive use and menopause after excluded females with history of hysterectomy. Models for postmenopausal breast was additionally adjusted for HRT and oral contraceptive use.

^a^Multivariable models were adjusted for sex, age, ethnicity, Townsend deprivation index, qualification, employment status, alcohol intaking, smoking status.

^b^Multivariable models were adjusted for sex, age, ethnicity, Townsend deprivation index, qualification, employment status, alcohol intaking, smoking status, metabolic status (hyperglycemia, low HDL cholesterol, hypertriglyceridemia, elevated BP).

The HRs and CIs of the associations of obesity status with and without MetS with 22 types of incident cancer are shown in Table 3. The MUO phenotype was associated with higher risks of 10 obesity-related cancers, as follows: oesophagus, stomach, colorectal, liver, pancreas, endometrium, biliary tract, postmenopausal breast, kidney and bladder cancers. However, the MUO phenotype was associated with a lower risk of prostate cancer. In contrast, the MHO phenotype was also associated with increased risks of five cancers: endometrium (HR = 2.78, 95% CI: 2.08–3.72), oesophagus (HR = 2.07, 95% CI: 1.32–3.23), kidney (HR = 1.71, 95% CI: 1.18–2.47), pancreas (HR = 1.61, 95% CI: 1.08–2.42) and postmenopausal breast (HR = 1.36, 95% CI: 1.16–1.59) cancers. We did not find that the risks of obesity-related cancers, including biliary tract, liver, bladder and colorectal cancers and multiple myeloma, were significantly increased among MHO individuals. In addition, the MH-OW phenotype was associated with higher risks of endometrium, pancreas and postmenopausal breast cancer. The MU-OW phenotype was associated with increased risks of endometrium, kidney, oesophagus, postmenopausal breast, colorectal and thyroid cancers. However, we did not find an independent association of the MU-NM phenotype with any of these site-specific cancers (Table 3).

**Table 3:** Multivariable hazard ratios and 95% confidence intervals of cancers in relation to overweight/obesity and MetS.

Cancer type/sites	Metabolically healthy			Metabolically unhealthy		
	Normal weight	Overweight	Obesity	Normal weight	Overweight	Obesity
Endometrium	1 (Ref.)	1.43 (1.09–1.85)	2.78 (2.08–3.72)	1.14 (0.81–1.61)	1.82 (1.41–2.36)	4.03 (3.20–5.08)
Kidney	1 (Ref.)	1.23 (0.92–1.65)	1.71 (1.18–2.47)	1.13 (0.80–1.60)	1.51 (1.16–1.97)	2.16 (1.66–2.81)
Biliary tract	1 (Ref.)	1.49 (0.66–3.33)	1.78 (0.64–4.96)	0.94 (0.34–2.61)	0.65 (0.27–1.55)	2.09 (1.00–4.44)
Liver	1 (Ref.)	0.95 (0.58–1.54)	1.32 (0.72–2.45)	1.51 (0.93–2.47)	1.24 (0.82–1.89)	1.87 (1.23–2.82)
Bladder	1 (Ref.)	1.18 (0.83–1.66)	1.27 (0.80–2.04)	1.37 (0.94–1.98)	1.35 (0.99–1.83)	1.75 (1.28–2.39)
Pancreas	1 (Ref.)	1.36 (1.01–1.85)	1.61 (1.08–2.42)	1.24 (0.87–1.76)	1.30 (0.98–1.73)	1.73 (1.29–2.32)
Esophagus	1 (Ref.)	1.19 (0.81–1.75)	2.07 (1.32–3.23)	1.38 (0.91–2.08)	1.61 (1.15–2.25)	1.61 (1.13–2.88)
Stomach	1 (Ref.)	1.33 (0.87–2.01)	0.78 (0.39–1.57)	1.07 (0.65–1.76)	1.31 (0.89–1.93)	1.51 (1.01–2.25)
Postmenopausal breast	1 (Ref.)	1.15 (1.02–1.29)	1.36 (1.16–1.59)	1.05 (0.91–1.21)	1.21 (1.08–1.36)	1.29 (1.14–1.46)
Colorectal	1 (Ref.)	1.12 (0.98–1.27)	1.10 (0.92–1.33)	1.13 (0.97–1.31)	1.19 (1.06–1.34)	1.29 (1.14–1.47)
Prostate	1 (Ref.)	0.96 (0.87–1.05)	0.90 (0.77–1.04)	0.93 (0.83–1.04)	0.97 (0.89–1.06)	0.79 (0.72–0.87)
Cervix	1 (Ref.)	1.43 (0.63–3.24)	1.01 (0.28–3.59)	2.24 (0.90–5.57)	1.64 (0.69–3.89)	2.18 (0.96–4.94)
Gallbladder	1 (Ref.)	1.24 (0.52–2.94)	0.63 (0.14–2.88)	0.79 (0.27–2.34)	1.02 (0.45–2.33)	1.31 (0.58–2.98)
Ovary	1 (Ref.)	1.16 (0.88–1.55)	1.16 (0.78–1.73)	1.20 (0.84–1.71)	1.06 (0.77–1.44)	1.26 (0.93–1.72)
Thyroid	1 (Ref.)	0.82 (0.51–1.31)	1.52 (0.89–2.62)	1.22 (0.71–2.08)	1.79 (1.20–2.67)	1.25 (0.79–1.96)
Oral	1 (Ref.)	0.86 (0.56–1.32)	0.91 (0.49–1.68)	1.32 (0.85–2.05)	0.99 (0.67–1.44)	1.21 (0.82–1.79)
Leukemia	1 (Ref.)	0.88 (0.66–1.17)	0.75 (0.48–1.16)	0.95 (0.68–1.31)	1.19 (0.93–1.53)	1.04 (0.80–1.36)
Non-Hodgkin lymphoma	1 (Ref.)	0.93 (0.75–1.14)	0.98 (0.72–1.32)	1.00 (0.78–1.27)	1.03 (0.85–1.25)	1.03 (0.84–1.26)
Malignant melanoma	1 (Ref.)	1.17 (0.99–1.38)	0.85 (0.65–1.12)	0.86 (0.69–1.08)	1.08 (0.92–1.27)	1.02 (0.85–1.23)
Multiple myeloma	1 (Ref.)	0.76 (0.54–1.07)	1.44 (0.96–2.16)	0.57 (0.36–0.90)	0.86 (0.63–1.17)	1.01 (0.73–1.40)
Lung	1 (Ref.)	0.86 (0.73–1.03)	0.97 (0.77–1.22)	1.19 (1.00–1.41)	1.00 (0.87–1.16)	0.90 (0.77–1.06)
Brain	1 (Ref.)	0.79 (0.57–1.11)	1.01 (0.64–1.58)	0.93 (0.63–1.36)	1.01 (0.75–1.36)	0.74 (0.53–1.05)

Multivariable models were adjusted for sex, age, ethnicity, Townsend deprivation index, qualification, employment status, alcohol intaking, smoking status. Models for cervix, ovary and endometrium cancers are additionally adjusted for HRT use, oral contraceptive use and menopause after excluded females with history of hysterectomy. Models for postmenopausal breast was additionally adjusted for HRT and oral contraceptive use.

In addition, we found that central obesity was also associated with eight incident cancers: oesophagus, colorectal, liver, pancreas, postmenopausal breast, endometrium, kidney and bladder cancers (Supplementary Table 3). Central obesity without MetS was only associated with increased risks of postmenopausal breast and endometrium cancer, but had no independent associations with any other central obesity-related cancers (Supplementary Table 4).

Upon exploring the associations between BMI and cancers stratified by metabolic status, we further confirmed that compared with metabolically healthy normal-weight individuals, MHO phenotype was associated with higher risks of cancers: endometrium, kidney, pancreas, postmenopausal breast and oesophagus cancers (Supplementary Table 5). Sensitivity analyses using a definition of metabolic status that includes WC with the ATP-III criterion showed consistent positive associations of MHO and specific cancers (Supplementary Table 6). We repeated the main analysis among participants with at least 2 years of follow-ups, and similar results were observed (Supplementary Table 7). Simultaneously, the results were generally consistent for sensitivity analyses that excluded individuals who had a history of cardiovascular disease or diabetes at baseline (Supplementary Table 8). The results regarding the associations of BMI and cancers, as well as those regarding the associations of BMI-metabolic factors and cancers with multiple imputed data showed that the HRs remained statistically significant. Obesity was consistently associated with higher risks of 10 of 22 cancers (Supplementary Table 9), among which the risks of five cancers, including oesophagus, pancreas, postmenopausal breast, endometrium and kidney cancers, remained significantly increased among those who were metabolically healthy (Supplementary Table 10).

## Discussion

In this prospective study of approximately 0.4 million adults, 6.7% of the included population were affected by obesity, among whom over a quarter were metabolically healthy. We found that obesity was independently associated with 12 of 22 cancers including endometrium, biliary tract, kidney, pancreas, oesophagus, liver, bladder, postmenopausal breast, colorectal, lung and prostate cancers and multiple myeloma. Our primary findings were that individuals with obesity, whether or not they were metabolically healthy, were at increased risks of oesophagus, pancreas, postmenopausal breast, endometrium and kidney cancers. However, we did not find that individuals with obesity and metabolically healthy were significantly associated with higher risks of other obesity-related cancers including biliary tract, liver, bladder, colorectal, stomach and prostate cancers.

Our study systematically explored the role of metabolic health in the associations of obesity with the full spectrum of cancer types. The risks of incident cancers according to BMI categories was evaluated separately in metabolically healthy and unhealthy phenotypes. The MHO phenotype had higher risks of oesophagus, pancreas, postmenopausal breast, endometrium and kidney cancers, and these risks remained for the MUO phenotype. This suggests that obesity outweighs the impact of metabolic health in the risks of these cancers. Although many studies have shown that BMI is associated with these cancers,^26^ these studies did not take into account the role of metabolic status. Being metabolically unhealthy may mask the risk of obesity for cancers. In accordance with previous evidence on certain types of cancer, many studies have shown that the MHO phenotype is associated with an increased risk of breast cancer.^15,17^ A study consisting of 50,884 participants in America suggested that postmenopausal women who are metabolically unhealthy or have central adiposity may have an increased risk for breast cancer despite having normal BMIs.^15^ Additionally, negative associations of obesity with lung and prostate cancers were observed in metabolically unhealthy individuals. Previous studies have suggested that obesity is inversely associated with decreased risks of lung and prostate cancers.^27,28^ The potential biological mechanism for the relationship between obesity and lung cancer is that leanness may be involved in the carcinogenic effect of smoking.^27^ It is possible that the negative association between BMI and prostate cancer might be due to detection bias since in one previous cohort, men with obesity were less likely to have undergone a prostate-specific antigen (PSA) test.^29^ Moreover, previous studies have reported slightly lower PSA levels in men with high BMI.^30^

However, in the present study, the patterns were different for colorectal, bladder, biliary tract, stomach and liver cancers. Specifically, although the MHO phenotype was not significantly associated with these cancers, increased risks of these cancers were found for the MUO phenotype. Thus, the MUO phenotype outweighed the effect of obesity on the risks of these cancers, as the higher risks of these cancers that are associated with general obesity may be explained by individuals being metabolically unhealthy. For these obesity-related cancers, the associations were stratified by metabolic status. Unfortunately, we did not have information about the trajectories of BMI and metabolic health and, therefore, could not distinguish between the contributing and confounding roles of metabolic status. It is currently recognised that a proportion of individuals with obesity may not be associated with a higher risk for metabolic complications. Recent studies have also examined the association of metabolic health with certain cancers.^16^ Therefore, the harmful effects of obesity on the incidence of several cancers may be partially offset by metabolic health. In our present study, however, we only observed that obesity increased risks of colorectal cancer in the metabolically unhealthy group, which is inconsistent with a previous Korean nested case-control study^13^ and a recent cohort study from the Women’s Health Initiative; these previous studies demonstrated that obesity increased the risk of colorectal cancer in individuals who were metabolically healthy.^14^ The association between MHO and thyroid cancer remains controversial, and a large cohort involving 255,051 participants in Korea showed that women with MUO, but not MHO, had an increased risk for thyroid cancer.^31^ However, our present study found no association between obesity and thyroid cancer in either metabolically healthy or metabolically unhealthy individuals.

In our present study, we demonstrated an independent association between central obesity and site-specific cancers. Our findings were supported by a recent large cohort study of 22.9 million adults, which revealed that central obesity, independent of general obesity, was associated with elevated risks of several cancers.^32^ A UK Biobank study also found a significant positive association between adiposity and breast cancer, and revealed that central obesity was associated with postmenopausal breast cancer.^33^ Our current study generated additional new evidence that the metabolically healthy central obesity phenotype was also associated with a higher risk of postmenopausal breast cancer, which may be interpreted as central obesity outweighing the impact of metabolic health on the risk of postmenopausal breast cancer.

Given the prevalence of the metabolic health among the obese population, emphasis has been increasingly placed on understanding the potential mechanisms underlying metabolic status. Obesity is characterised by an excess of adipose tissue and commonly accompanies metabolic deterioration, such as hyperglycaemia, hypertension, dyslipidaemia and insulin resistance.^11^ Indeed, disruptions in insulin metabolism, adipokines, inflammation and sex hormones all contribute to the adverse effects of obesity in cancer development and progression.^34^ MetS is also associated with increased risks of various cancers.

It has been suggested that hormones play a role in the maintenance of a healthy status in some individuals with obesity, and their geographical location, metabolic activities and histological characteristics of these individuals may partly determine their metabolic health.^35^ Although MHO individuals accumulate high body fat, they display a high concentration of insulin sensitivity and lower C-reactive protein and high adiponectin concentrations.^36^ Further evidence has shown that MHO individuals are more fit than their metabolically unhealthy counterparts.^37^ This favourable profile might reduce their risks of certain types of obesity-related cancers, such as liver and bladder cancers. Several previous studies have also shown that a subgroup of individuals with obesity might be protected from metabolic obesity-related complications or might be at a substantially lower risk than expected based on their degrees of obesity.^11^

Notable strengths of the present study included its prospective design and its relatively large sample size, which provided us with modest statistical power to assess the association of BMI-metabolic status with multiple cancers. In addition, we conducted a series of sensitivity analyses, all of which yielded qualitatively similar results, indicating the robustness of our findings. This is the first study, to the best of our knowledge, to investigate the associations between obesity with and without MetS and the risks of cancers. Our findings may help to determine the importance of obesity and metabolic status for the prevention of specific cancers. Despite these strengths, several limitations of our study need to be considered. First, our definition of MHO was not consistent with that used for many previous studies.^7,25^ Moreover, by definition, metabolic status gives a simplified picture of diverse and complex phenotypes. To overcome this limitation, we selected the most common definition (based on ATP- III components) and compared several alternative definitions in our sensitivity analyses, all of which yielded qualitatively similar results. Second, we used BMI as a marker of obesity, but BMI does not differentiate fat tissue from lean tissue. If the individuals with the MHO phenotype had a higher proportion of lean mass than those with the normal-weight phenotype, and the associations between MHO and the risks of cancers in our present study may have been attenuated. Third, although we adjusted for potential confounders, other unmeasured and residual confounding factors that we did not assess may have influenced the associations that we reported. Additionally, an observational study such as ours cannot fully infer casual associations. Finally, BMI or metabolic status can change over time in a substantial proportion of the population; however, our study did not reflect longitudinal changes in body weights or laboratory findings.

## Conclusion

The present study found that obesity was independently associated with higher risks of 10 of 22 types of cancer. Even in metabolically healthy individuals, obesity was associated with increased risks of five types of cancer, although these individuals were not significantly associated with risks of other obesity-related cancers. Our findings support that MHO does not appear to be a benign condition for several cancers, making it important to promote prudent weight loss in individuals with obesity regardless of their initial metabolic status. In regard to weight management, other healthy behaviours and lifestyle factors should be implemented to increase the likelihood of one transitioning from the MUO to MHO phenotype, thus reducing the risk for obesity-related cancers.

## Supplementary information

Supplementary material

## Data Availability

Information about data access is available online (http://www.ukbiobank.ac.uk/wp-content/uploads/2011/11/UK-Biobank-Protocol.pdf)
