# Supplementary material for: Association of obesity status and metabolic syndrome with site-specific cancers: a population-based cohort study
Source: Br J Cancer. 2020 Jul 30;123(8):1336–44. doi: 10.1038/s41416-020-1012-6 (PMC7555864; doi:10.1038/s41416-020-1012-6)
Supplement: Supplementary file 1 — Supplementary material [file 41416_2020_1012_MOESM1_ESM.doc]

**Supplementary material**

**Detailed methodology**

Sociodemographic characteristics

Participants provided detailed self-reported data via a touch screen questionnaire at the assessment centres at baseline. Socio-demographic factors included age at recruitment (month and year of birth as acquired by the central registry and updated by participant, with each participant nominally assigned 15 as the day of birth), ethnicity (participants were asked “What is your ethnic group?” with possible answers being “White”, “Mixed”, “Asian or Asian British”, “Black or “Black British”, “Chinese”, “Other ethnic group”, “Do not know”, “Prefer not to answer”, qualifications (education attainment) (“Which of the following qualifications do you have?” with possible answers being “College degree”, “A levels/AS levels”, “O levels/GCESs”, “CSEs”, “NVQ or HND or HNC”, “Other professional qualifications”, “Do not know”, “Prefer not to answer”), employment (possible answers classify as “Working”, “retired”, “unemployment”, “other”), Townsend deprivation index was assigned based on postcode as a continuous measure, which derived from census data on housing, employment, social class and car availability, a higher index indicates more deprivation.

Anthropometric measurements

Anthropometric measurements were taken by trained research clinic staff (UK-Biobank, 2014). Standing height was measured to the nearest centimetre (cm), without shoes, using the SECA 240 Height Measure. Weight measurement was measured without shoes and outdoor clothing, using the Tanita BC 418 body composition analyser or using standard scales if the participant did not undergo bioimpedance analysis. Body mass index (BMI) was calculated as weight (kg)/height (m^2^). Systolic/diastolic blood pressure were calculated from the second measurement of the two baseline recordings.

Lifestyle characteristics

Smoking status was self-reported, and based on current/past smoking status the participant was classified as never, former, or current smoker.

Alcohol intake was asked participants how often they drank alcohol with the possible answers being: “daily or almost daily”, “three or four times a week”, “once or twice a week”, “one to three times a month”, “special occasions only”, “never”, “prefer not to answer”. If the participant reported ‘do not know’ or ‘prefer not to answer’ to one of these questions on weekly or monthly consumption, they were coded as missing.

Health status

Most medical conditions and operations were identified in a verbal interview with a trained research clinic staff, in which vasectomy status was recorded. We used participants’ answers to the question “Has a doctor ever told you that you have diabetes?” to identify participants with diabetes. We used participants’ answers to the question “Has a doctor ever told you that you have CVD (Heart attack, angina, stroke)?” to identify participants with CVD. Menopause was identified by the question “Have you had your menopause (periods stopped)”. Hormone Replacement Therapy was attained by the question “Have you ever used hormone replacement therapy (HRT)”. Hysterectomy was attained by the question “Have you had a hysterectomy (womb removed)”. The information of medication for blood pressure, cholesterol, diabetes was acquired by the question “Do you regularly take any of the following medications?” The response includes cholesterol lowering medication, blood pressure medication and insulin.

Further details of these measurements can be found in the UK Biobank online protocol (http://www.ukbiobank.ac.uk).

**Table 1: ICD-10 code of 22 types of cancer.**

| Cancer type/sites | ICD-10 code |
| --- | --- |
| Oral | C00, C01, C02, C03, C04, C05, C06 |
| Esophagus | C15 |
| Stomach | C16 |
| Colorectal | C18, C19, C20 |
| Liver | C22 |
| Gallbladder | C23 |
| Biliary tract | C24 |
| Pancreas | C25 |
| Lung | C34 |
| Malignant melanoma | C43 |
| Breast | C50 |
| Cervix | C53 |
| Endometrium | C54 |
| Ovary | C56 |
| Prostate | C61 |
| Kidney | C64, C65 |
| Bladder | C67 |
| Brain | C71 |
| Thyroid | C73 |
| Non-Hodgkin lymphoma | C82, C83, C84, C85 |
| Multiple myeloma | C90 |
| Leukemia | C91, C92, C93, C94, C95 |

**Table 2: Baseline characteristics of participants across BMI.**

| Characteristics | Total | BMI | | |
| --- | --- | --- | --- | --- |
|  | (390 575) | Normal weight (126 857) | Overweight  (167 545) | Obesity  (96 173) |
| Female | 206954 (53.0) | 81158 (64.0) | 76514(45.7) | 49282(51.2) |
| Age | 56.3 (8.1) | 55.4 (8.2) | 56.7(8.1) | 56.6(7.9) |
| Ethnicity |  |  |  |  |
| White | 367393 (94.1) | 119918 (94.5) | 157688(94.1) | 89787(93.4) |
| Mixed background | 2339 (0.6) | 825 (0.7) | 917(0.6) | 597(0.6) |
| South Asian | 7834 (2.0) | 2550 (2.0) | 3610(2.2) | 1674(1.7) |
| Black | 6340 (1.6) | 1200 (1.0) | 2630(1.6) | 2510(2.6) |
| Chinese | 1231 (0.3) | 778 (0.6) | 388(0.2) | 65(0.1) |
| Others | 3599 (0.9) | 1076 (0.8) | 1520(0.9) | 1003(1.0) |
| Townsend deprivation index |  |  |  |  |
| 1 (least deprived) | 78572 (20.1) | 27469 (21.7) | 35019(20.9) | 16084(16.7) |
| 2 | 78041 (20.0) | 26243 (20.1) | 34498(20.6) | 17300(18.0) |
| 3 | 77525 (19.8) | 25216 (20.0) | 33961(20.3) | 18348(19.1) |
| 4 | 78556 (20.1) | 25067 (19.8) | 33190(19.8) | 20299(21.1) |
| 5 (most deprived) | 77404 (19.8) | 22716 (17.9) | 30671(18.3) | 24017(25.0) |
| Employment |  |  |  |  |
| Working | 229089 (58.7) | 77541 (61.1) | 97691(58.3) | 53857(56.0) |
| Retired | 124975 (32.0) | 37495 (29.6) | 56525(33.7) | 30955(32.2) |
| Unemployed | 29487 (7.6) | 9325 (7.4) | 10503(6.3) | 9659(10.0) |
| Other | 7024 (1.8) | 2496 (2.0) | 2826(1.7) | 1702(1.8) |
| Qualifications |  |  |  |  |
| College degree | 127067 (32.5) | 50595 (40.0) | 52893(31.6) | 23579(24.5) |
| A levels/AS levels | 43588 (11.2) | 15390 (12.1) | 18269(10.9) | 9929(10.3) |
| O levels/GCESs | 82516 (21.1) | 25716 (20.3) | 35727(21.3) | 21073(21.9) |
| CSEs | 21469 (5.5) | 6075 (4.8) | 9132(5.5) | 6262(6.5) |
| NVQ or HND or HNC | 25833 (6.6) | 6231 (4.9) | 11971(7.1) | 7631(7.9) |
| Other professional qualifications | 20014 (5.1) | 5984 (4.7) | 8682(5.2) | 5348(5.6) |
| None of the above | 65480 (16.8) | 15681 (12.4) | 28884(17.2) | 20915(21.8) |
| Smoking status |  |  |  |  |
| Never | 213724 (54.7) | 74977 (59.1) | 89676(53.5) | 49071(51.0) |
| Previous | 133744 (34.2) | 36979 (29.2) | 59687(35.6) | 37078(38.6) |
| Current | 41173 (10.5) | 14416 (11.4) | 17350(10.4) | 9407(9.8) |
| Alcohol intake frequency |  |  |  |  |
| Daily or almost daily | 79429 (20.3) | 28643 (22.6) | 36076(21.5) | 14710(15.3) |
| Three or four times a week | 90723 (23.2) | 31301 (24.7) | 40975(24.5) | 18447(19.2) |
| Once or twice a week | 100933 (25.8) | 31971 (25.2) | 43734(26.1) | 25228(26.2) |
| One to three times a month | 43480 (11.1) | 13072 (10.3) | 17534(10.5) | 12874(13.4) |
| Special occasions only | 44268 (11.3) | 12504 (9.9) | 16891(10.1) | 14873(15.5) |
| Never | 30894 (7.9) | 9122 (7.2) | 12000(7.2) | 9772(10.2) |
| Metabolic healthy | 196285(50.3) | 36347(28.7) | 89859(53.6) | 70079(72.9) |
| Hyperglycemia | 59363(15.2) | 22112(23.0) | 24055(14.4) | 13196(10.4) |
| Low HDL-cholesterol | 78685(20.2) | 12812(10.1) | 32005(19.1) | 33868(35.2) |
| Hypertriglyceridemia | 190613(48.8) | 36122(28.5) | 89218(53.3) | 65273(67.9) |
| Elevated blood pressure | 281539 (72.1) | 73828 (58.2) | 125731 (75.0) | 81980 (85.2) |

Note: a Values are numbers (percentages) unless stated otherwise

Abbreviations: BMI, body mass index (calculated as weight in kilograms divided by height in meters squared); BP, blood pressure; CSE, Certificate of Secondary Education; GCSE, General Certificate of Secondary Education; HNC, Higher National Certificate; HND, Higher National Diploma; NVQ, National Vocational Qualification.

**Table 3: Multivariable HRs and 95% CIs of cancers in relation to central obesity.**

| Cancer type/sites | Normal weight | Central obesity | |
| --- | --- | --- | --- |
|  |  | Unadjusted ^a^ | Adjusted ^b^ |
| Oral | 1 (Ref.) | 0.81 (0.63-1.04) | 0.77 (0.59-1.00) |
| Esophagus | 1 (Ref.) | **1.35 (1.09-1.66)** | **1.29 (1.04-1.60)** |
| Stomach | 1 (Ref.) | 1.22 (0.95-1.57) | 1.16 (0.89-1.50) |
| Colorectal | 1 (Ref.) | **1.23 (1.14-1.34)** | **1.20 (1.10-1.31)** |
| Liver | 1 (Ref.) | **1.44 (1.10-1.89)** | 1.31 (0.98-1.74) |
| Biliary tract | 1 (Ref.) | 1.41 (0.84-2.37) | 1.35 (0.78-2.31) |
| Gallbladder | 1 (Ref.) | 1.31 (0.73-2.34) | 1.28 (0.69-2.36) |
| Pancreas | 1 (Ref.) | 1.23 (1.03-1.49) | 1.17 (0.96-1.42) |
| Lung | 1 (Ref.) | 1.06 (0.96-1.17) | 1.03 (0.93-1.15) |
| Malignant melanoma | 1 (Ref.) | 1.10 (0.98-1.23) | **1.14 (1.01-1.28)** |
| Postmenopausal Breast | 1 (Ref.) | **1.30 (1.19-1.41)** | **1.29 (1.18-1.41)** |
| Cervix | 1 (Ref.) | 0.87 (0.51-1.49) | 0.67 (0.37-1.20) |
| Endometrium | 1 (Ref.) | **2.16 (1.80-2.58)** | **1.82 (1.51-2.20)** |
| Ovary | 1 (Ref.) | 1.07 (0.87-1.32) | 1.05 (0.84-1.30) |
| Prostate | 1 (Ref.) | 0.98 (0.93-1.04) | 1.00 (0.94-1.06) |
| Kidney | 1 (Ref.) | **1.53 (1.29-1.82)** | **1.39 (1.16-1.66)** |
| Bladder | 1 (Ref.) | **1.28 (1.06-1.55)** | 1.20 (0.99-1.47) |
| Brain | 1 (Ref.) | 0.84 (0.68-1.04) | 0.84 (0.67-1.04) |
| Thyroid | 1 (Ref.) | 1.09 (0.82-1.45) | 0.96 (0.71-1.30) |
| Non-Hodgkin lymphoma | 1 (Ref.) | 1.14 (1.00-1.30) | 1.11 (0.96-1.28) |
| Multiple myeloma | 1 (Ref.) | 1.14 (0.92-1.42) | 1.19 (0.94-1.49) |
| Leukemia | 1 (Ref.) | 1.04 (0.87-1.23) | 0.95 (0.79-1.13) |

^a^ Multivariable models were adjusted for sex, age, ethnicity, Townsend deprivation index, qualification, employment status, alcohol intaking, smoking status.

^b^ Multivariable models were adjusted for sex, age, ethnicity, Townsend deprivation index, qualification, employment status, alcohol intaking, smoking status, metabolic status (hyperglycemia, low HDL-cholesterol, hypertriglyceridemia, elevated BP).

Models for cervix, ovary and endometrium cancers are additionally adjusted for HRT use, oral contraceptive use and menopause after excluded females with history of hysterectomy. Models for postmenopausal breast was additionally adjusted for HRT and oral contraceptive use.

**Table 4: Multivariable hazard ratios and 95% confidence intervals of cancers in relation to central obesity and MetS.**

| Cancer type/sites | Metabolically healthy | |  | Metabolically unhealthy | |
| --- | --- | --- | --- | --- | --- |
|  | Normal weight | Central obesity |  | Normal weight | Central obesity |
| Oral | 1 (Ref.) | 0.72 (0.30-1.73) |  | 1.21 (0.72-2.02) | 0.96 (0.58-1.59) |
| Esophagus | 1 (Ref.) | 1.61 (0.68-3.78) |  | 1.72 (0.92-3.20) | **2.21 (1.20-4.05)** |
| Stomach | 1 (Ref.) | 0.53 (0.19-1.43) |  | 0.79 (0.46-1.34) | 1.02 (0.61-1.70) |
| Colorectal | 1 (Ref.) | 1.21 (0.95-1.54) |  | 0.97 (0.82-1.16) | **1.20 (1.02-1.42)** |
| Liver | 1 (Ref.) | 1.20 (0.46-3.15) |  | 1.14 (0.58-2.24) | 1.64 (0.86-3.12) |
| Biliary tract | 1 (Ref.) | 3.30 (0.30-36.45) |  | 3.67 (0.49-27.62) | 4.64 (0.64-33.91) |
| Gallbladder | 1 (Ref.) | 0.52 (0.05-5.04) |  | 0.93 (0.26-3.31) | 1.28 (0.39-4.21) |
| Pancreas | 1 (Ref.) | 0.74 (0.39-1.38) |  | 0.84 (0.57-1.24) | 1.09 (0.76-1.57) |
| Lung | 1 (Ref.) | 0.93 (0.65-1.33) |  | 1.10 (0.87-1.40) | 1.16 (0.92-1.46) |
| Malignant melanoma | 1 (Ref.) | 1.07 (0.80-1.44) |  | 0.83 (0.67-1.02) | 0.94 (0.77-1.15) |
| Postmenopausal Breast | 1 (Ref.) | **1.25 (1.01-1.55)** |  | 1.10 (0.93-1.30) | **1.41 (1.21-1.64)** |
| Cervix | 1 (Ref.) | 0.18 (0.02-1.42) |  | 0.77 (0.34-1.76) | 0.82 (0.39-1.74) |
| Endometrium | 1 (Ref.) | **1.64 (1.01-2.65)** |  | **1.51 (1.03-2.22)** | **3.12 (2.20-4.44)** |
| Ovary | 1 (Ref.) | 1.12 (0.69-1.83) |  | 1.20 (0.83-1.73) | 1.24 (0.88-1.75) |
| Prostate | 1 (Ref.) | 1.18 (0.94-1.48) |  | 1.06 (0.92-1.22) | 1.03 (0.89-1.18) |
| Kidney | 1 (Ref.) | 1.69 (0.92-3.12) |  | 1.57 (0.99-2.48) | **2.30 (1.48-3.58)** |
| Bladder | 1 (Ref.) | 1.38 (0.71-2.66) |  | 1.01 (0.63-1.63) | 1.29 (0.82-2.03) |
| Brain | 1 (Ref.) | 1.30 (0.73-2.31) |  | 1.10 (0.72-1.67) | 0.88 (0.59-1.32) |
| Thyroid | 1 (Ref.) | 0.77 (0.35-1.68) |  | 1.07 (0.64-1.79) | 1.19 (0.74-1.92) |
| Non-Hodgkin lymphoma | 1 (Ref.) | 1.13 (0.77-1.65) |  | 0.90 (0.69-1.18) | 1.04 (0.81-1.35) |
| Multiple myeloma | 1 (Ref.) | 1.60 (0.83-3.08) |  | 1.25 (0.76-2.06) | 1.37 (0.85-2.21) |
| Leukemia | 1 (Ref.) | 0.83 (0.47-1.49) |  | 1.01 (0.70-1.46) | 1.06 (0.75-1.51) |

Multivariable models were adjusted for sex, age, ethnicity, Townsend deprivation index, qualification, employment status, alcohol intaking, smoking status. Models for cervix, ovary and endometrium cancers are additionally adjusted for HRT use, oral contraceptive use and menopause after excluded females with history of hysterectomy. Models for postmenopausal breast was additionally adjusted for HRT and oral contraceptive use.

**Table 5: Multivariable hazard ratios and 95% confidence intervals of cancers according to BMI categories stratified by metabolic status.**

| Cancer type/sites | Metabolically healthy | | | | Metabolically unhealthy | | | |
| --- | --- | --- | --- | --- | --- | --- | --- | --- |
|  | Normal weight | Overweight | Obesity | *P* for trend | Normal weight | Overweight | Obesity | *P* for trend |
| Endometrium | 1 (Ref.) | **1.42 (1.09-1.85)** | **2.79 (2.08-3.75)** | <0.001 | 1 (Ref.) | **1.60 (1.15-2.24)** | **3.52 (2.56-4.83)** | <0.001 |
| Kidney | 1 (Ref.) | 1.16 (0.86-1.56) | **1.64 (1.13-2.37)** | 0.012 | 1 (Ref.) | **1.38 (1.02-1.86)** | **1.95 (1.44-2.64)** | <0.001 |
| Pancreas | 1 (Ref.) | 1.30 (0.95-1.76) | **1.58 (1.05-2.38)** | 0.019 | 1 (Ref.) | 1.08 (0.79-1.49) | **1.41 (1.02-1.95)** | 0.012 |
| Postmenopausal Breast | 1 (Ref.) | **1.14 (1.01-1.28)** | **1.35 (1.15-1.58)** | <0.001 | 1 (Ref.) | **1.16 (1.01-1.34)** | **1.24 (1.06-1.44)** | <0.001 |
| Oral | 1 (Ref.) | 0.85 (0.56-1.31) | 0.87 (0.47-1.62) | 0.592 | 1 (Ref.) | 0.75 (0.50-1.12) | 0.93 (0.61-1.41) | 0.838 |
| Esophagus | 1 (Ref.) | 1.26 (0.85-1.85) | **2.07 (1.32-3.26)** | 0.002 | 1 (Ref.) | 1.13 (0.81-1.58) | 1.16 (0.81-1.64) | 0.454 |
| Stomach | 1 (Ref.) | 1.33 (0.87-2.03) | 0.79 (0.39-1.61) | 0.89 | 1 (Ref.) | 1.23 (0.80-1.90) | 1.41 (0.90-2.20) | 0.123 |
| Colorectal | 1 (Ref.) | 1.13 (0.99-1.29) | 1.11 (0.92-1.34) | 0.128 | 1 (Ref.) | 1.04 (0.91-1.20) | 1.13 (0.98-1.31) | 0.035 |
| Liver | 1 (Ref.) | 1.00 (0.61-1.64) | 1.35 (0.73-2.52) | 0.38 | 1 (Ref.) | 0.81 (0.53-1.22) | 1.24 (0.82-1.87) | 0.067 |
| Gallbladder | 1 (Ref.) | 1.31 (0.55-3.12) | 0.62 (0.13-2.86) | 0.755 | 1 (Ref.) | 1.25 (0.45-3.48) | 1.70 (0.60-4.74) | 0.257 |
| Biliary tract | 1 (Ref.) | 1.46 (0.65-3.28) | 2.01 (0.72-5.63) | 0.167 | 1 (Ref.) | 0.68 (0.25-1.84) | 2.00 (0.81-4.94) | 0.010 |
| Lung | 1 (Ref.) | 0.87 (0.74-1.04) | 0.99 (0.78-1.25) | 0.648 | 1 (Ref.) | 0.84 (0.72-0.97) | 0.74 (0.63-0.88) | 0.001 |
| Malignant melanoma | 1 (Ref.) | **1.20 (1.01-1.41)** | 0.87 (0.66-1.14) | 0.889 | 1 (Ref.) | 1.23 (0.99-1.54) | 1.18 (0.94-1.50) | 0.334 |
| Cervix | 1 (Ref.) | 1.44 (0.63-3.31) | 1.04 (0.29-3.76) | 0.745 | 1 (Ref.) | 0.72 (0.28-1.84) | 0.95 (0.38-2.35) | 0.798 |
| Ovary | 1 (Ref.) | 1.13 (0.85-1.51) | 1.12 (0.75-1.67) | 0.451 | 1 (Ref.) | 0.89 (0.61-1.30) | 1.06 (0.73-1.55) | 0.48 |
| Prostate | 1 (Ref.) | 0.95 (0.87-1.05) | 0.89 (0.77-1.04) | 0.151 | 1 (Ref.) | 1.04 (0.94-1.14) | **0.85 (0.76-0.95)** | <0.001 |
| Bladder | 1 (Ref.) | 1.19 (0.84-1.68) | 1.25 (0.78-2.01) | 0.287 | 1 (Ref.) | 0.98 (0.72-1.33) | 1.28 (0.94-1.75) | 0.026 |
| Brain | 1 (Ref.) | 0.79 (0.56-1.10) | 1.00 (0.64-1.58) | 0.688 | 1 (Ref.) | 1.11 (0.77-1.60) | 0.81 (0.54-1.22) | 0.164 |
| Thyroid | 1 (Ref.) | 0.81 (0.50-1.30) | 1.48 (0.85-2.56) | 0.286 | 1 (Ref.) | 1.48 (0.89-2.46) | 1.04 (0.60-1.81) | 0.706 |
| Non-Hodgkin lymphoma | 1 (Ref.) | 0.91 (0.74-1.12) | 0.96 (0.71-1.30) | 0.652 | 1 (Ref.) | 1.04 (0.83-1.31) | 1.04 (0.81-1.32) | 0.754 |
| Multiple myeloma | 1 (Ref.) | 0.75 (0.53-1.07) | 1.50 (0.99-2.26) | 0.194 | 1 (Ref.) | 1.50 (0.96-2.34) | **1.69 (1.07-2.67)** | 0.040 |
| Leukemia | 1 (Ref.) | 0.88 (0.66-1.17) | 0.77 (0.50-1.20) | 0.211 | 1 (Ref.) | 1.25 (0.93-1.69) | 1.07 (0.78-1.48) | 0.905 |

Multivariable models were adjusted for sex, age, ethnicity, Townsend deprivation index, qualification, employment status, alcohol intaking, smoking status. Models for cervix, ovary and endometrium cancers are additionally adjusted for HRT use, oral contraceptive use and menopause after excluded females with history of hysterectomy. Models for postmenopausal breast was additionally adjusted for HRT and oral contraceptive use.

**Table 6: Multivariable hazard ratios and 95% confidence intervals for cancer across BMI and metabolic status where the definition of metabolic status includes the waist circumference criterion.**

| Cancer type/sites | Metabolically healthy | | |  | Metabolically unhealthy | | |
| --- | --- | --- | --- | --- | --- | --- | --- |
|  | Normal weight | Overweight | Obesity |  | Normal weight | Overweight | Obesity |
| Oral | 1 (Ref.) | 0.80 (0.56-1.15) | 0.81 (0.45-1.46) |  | 1.07 (0.59-1.94) | 0.88 (0.62-1.26) | 1.10 (0.77-1.55) |
| Esophagus | 1 (Ref.) | 1.13 (0.83-1.55) | **1.82 (1.21-2.76)** |  | 1.24 (0.74-2.08) | **1.47 (1.10-1.96)** | **1.45 (1.07-1.96)** |
| Stomach | 1 (Ref.) | 1.27 (0.88-1.83) | 0.78 (0.39-1.54) |  | 1.30 (0.70-2.39) | 1.41 (0.99-2.02) | **1.55 (1.08-2.23)** |
| Colorectal | 1 (Ref.) | 1.07 (0.96-1.20) | 1.08 (0.90-1.29) |  | 1.21 (1.00-1.47) | **1.22 (1.09-1.36)** | **1.28 (1.14-1.44)** |
| Liver | 1 (Ref.) | 0.68 (0.45-1.04) | 1.08 (0.61-1.92) |  | 0.99 (0.52-1.90) | 1.16 (0.81-1.67) | **1.55 (1.09-2.20)** |
| Biliary tract | 1 (Ref.) | 1.48 (0.72-3.04) | 1.85 (0.70-4.91) |  | 1.16 (0.33-4.09) | 0.59 (0.24-1.45) | **2.22 (1.12-4.40)** |
| Gallbladder | 1 (Ref.) | 1.14 (0.52-2.51) | 0.64 (0.14-2.84) |  | 0.69 (0.16-3.10) | 1.14 (0.53-2.46) | 1.36 (0.63-2.90) |
| Pancreas | 1 (Ref.) | **1.36 (1.04-1.79)** | **1.67 (1.14-2.46)** |  | **1.74 (1.16-2.61)** | **1.35 (1.03-1.77)** | **1.77 (1.35-2.32)** |
| Lung | 1 (Ref.) | 0.83 (0.71-0.96) | 0.94 (0.76-1.18) |  | 1.23 (1.00-1.51) | 1.00 (0.87-1.14) | 0.87 (0.76-1.00) |
| Malignant melanoma | 1 (Ref.) | **1.22 (1.05-1.42)** | 0.89 (0.68-1.16) |  | 1.01 (0.75-1.37) | 1.12 (0.95-1.32) | 1.07 (0.90-1.28) |
| Postmenopausal Breast | 1 (Ref.) | **1.15 (1.03-1.29)** | **1.37 (1.18-1.60)** |  | 1.15 (0.96-1.38) | **1.24 (1.10-1.39)** | **1.30 (1.16-1.46)** |
| Cervix | 1 (Ref.) | 1.08 (0.50-2.31) | 0.82 (0.24-2.81) |  | 1.41 (0.41-4.87) | 1.45 (0.65-3.26) | 1.77 (0.83-3.75) |
| Endometrium | 1 (Ref.) | **1.38 (1.08-1.77)** | **2.75 (2.08-3.64)** |  | 1.15 (0.74-1.79) | **1.86 (1.45-2.38)** | **3.95 (3.18-4.91)** |
| Ovary | 1 (Ref.) | 1.16 (0.89-1.52) | 1.13 (0.77-1.68) |  | 1.26 (0.81-1.97) | 0.99 (0.73-1.36) | 1.24 (0.92-1.66) |
| Prostate | 1 (Ref.) | 0.98 (0.90-1.06) | 0.95 (0.82-1.09) |  | 1.04 (0.89-1.22) | 1.02 (0.94-1.10) | 0.82 (0.75-0.90) |
| Kidney | 1 (Ref.) | **1.31 (1.02-1.69)** | **1.79 (1.26-2.53)** |  | 1.45 (0.95-2.19) | **1.54 (1.20-1.96)** | **2.18 (1.72-2.77)** |
| Bladder | 1 (Ref.) | 1.03 (0.77-1.38) | 1.16 (0.74-1.82) |  | 1.44 (0.92-2.25) | **1.34 (1.02-1.75)** | **1.64 (1.25-2.14)** |
| Brain | 1 (Ref.) | 0.91 (0.68-1.21) | 1.00 (0.65-1.55) |  | 0.90 (0.52-1.55) | 0.95 (0.71-1.27) | 0.75 (0.54-1.04) |
| Thyroid | 1 (Ref.) | 0.98 (0.65-1.49) | 1.62 (0.96-2.73) |  | 1.70 (0.90-3.20) | **1.78 (1.20-2.64)** | 1.24 (0.80-1.91) |
| Non-Hodgkin lymphoma | 1 (Ref.) | 0.91 (0.76-1.09) | 0.94 (0.70-1.26) |  | 0.84 (0.59-1.19) | 1.02 (0.85-1.23) | 1.01 (0.83-1.22) |
| Multiple myeloma | 1 (Ref.) | 0.83 (0.61-1.13) | **1.59 (1.07-2.35)** |  | 0.48 (0.23-0.98) | 0.96 (0.71-1.30) | 1.10 (0.81-1.49) |
| Leukemia | 1 (Ref.) | 0.90 (0.71-1.15) | 0.74 (0.48-1.13) |  | 0.90 (0.57-1.41) | 1.24 (0.99-1.56) | 1.05 (0.82-1.35) |

Multivariable models were adjusted for sex, age, ethnicity, Townsend deprivation index, qualification, employment status, alcohol intaking, smoking status. Models for cervix, ovary and endometrium cancers are additionally adjusted for HRT use, oral contraceptive use and menopause after excluded females with history of hysterectomy. Models for postmenopausal breast was additionally adjusted for HRT and oral contraceptive use.

**Table 7: Multivariable hazard ratios and 95% confidence intervals for cancer across BMI and metabolic status after exclusion of first two years of follow-up.**

| Cancer type/sites | Metabolically healthy | | |  | Metabolically unhealthy | | |
| --- | --- | --- | --- | --- | --- | --- | --- |
|  | Normal weight | Overweight | Obesity |  | Normal weight | Overweight | Obesity |
| Oral | 1 (Ref.) | 0.95 (0.60-1.51) | 1.00 (0.51-1.95) |  | 1.24 (0.75-2.06) | 0.92 (0.60-1.43) | 1.29 (0.24-2.02) |
| Esophagus | 1 (Ref.) | 1.26 (0.80-1.98) | **2.32 (1.39-2.89)** |  | 1.54 (0.95-2.48) | **1.81 (1.22-2.69)** | **1.91 (1.27-2.90)** |
| Stomach | 1 (Ref.) | 1.26 (0.79-2.02) | 0.80 (0.37-1.76) |  | 1.00 (0.57-1.76) | 1.23 (0.79-1.89) | 1.52 (0.96-2.39) |
| Colorectal | 1 (Ref.) | 1.13 (0.97-1.30) | 1.12 (0.90-1.38) |  | 1.04 (0.87-1.04) | 1.14 (1.00-1.31) | **1.35 (1.17-1.56)** |
| Liver | 1 (Ref.) | 1.17 (0.77-1.79) | 1.08 (0.59-1.95) |  | 1.06 (0.80-1.99) | 1.26 (0.66-1.46) | **1.50 (1.01-2.21)** |
| Biliary tract | 1 (Ref.) | 1.37 (0.63-2.98) | 1.04 (0.31-3.65) |  | 1.01 (0.97-1.05) | 0.59 (0.25-1.39) | **2.08 (1.05-4.12)** |
| Gallbladder | 1 (Ref.) | 1.26 (0.48-3.29) | 0.37 (0.05-2.95) |  | 0.72 (0.21-2.15) | 0.83 (0.32-2.15) | 1.19 (0.47-3.04) |
| Pancreas | 1 (Ref.) | 1.38 (0.98-1.94) | **1.63 (1.04-2.55)** |  | 1.26 (0.85-1.87) | 1.37 (0.99-1.89) | **1.80 (1.29-2.51)** |
| Lung | 1 (Ref.) | 0.85 (0.70-1.02) | 0.84 (0.64-1.10) |  | 1.05 (0.87-1.28) | 0.89 (0.76-1.05) | 0.83 (0.70-0.99) |
| Malignant melanoma | 1 (Ref.) | 1.16 (0.95-1.40) | 0.80 (0.58-1.11) |  | 0.89 (0.69-1.16) | 1.10 (0.91-1.34) | 1.07 (0.86-1.32) |
| Postmenopausal Breast | 1 (Ref.) | 1.06 (0.96-1.17) | **1.08 (1.12-1.46)** |  | 1.00 (0.88-1.15) | **1.19 (1.07-1.32)** | **1.25 (1.12-1.39)** |
| Cervix | 1 (Ref.) | 1.42 (0.60-3.42) | 0.86 (0.19-3.86) |  | 1.03 (0.89-1.15) | 1.21 (0.45-3.25) | 1.53 (0.59-4.00) |
| Endometrium | 1 (Ref.) | **1.56 (1.15-2.12)** | **2.82 (2.00-2.97)** |  | 1.16 (0.77-1.73) | **1.78 (1.31-2.40)** | **3.68 (2.28-4.87)** |
| Ovary | 1 (Ref.) | 1.10 (0.81-1.51) | 1.08 (0.70-1.69) |  | 0.87 (0.57-1.69) | 1.21 (0.88-1.67) | 1.17 (0.84-1.65) |
| Prostate | 1 (Ref.) | 1.00 (0.90-1.12) | 0.95 (0.80-1.12) |  | 0.99 (0.87 -1.13) | 1.02 (0.92-1.12) | **0.85 (0.76-0.96)** |
| Kidney | 1 (Ref.) | 1.28 (0.93-1.77) | **1.55 (1.02-2.36)** |  | 0.96 (0.64-1.43) | **1.38 (1.03-1.85)** | **2.07 (1.53-2.78)** |
| Bladder | 1 (Ref.) | 1.12 (0.75-1.67) | 1.63 (0.98-2.70) |  | 1.25 (0.81-1.92) | 1.24 (0.87-1.77) | **1.75 (1.22-2.53)** |
| Brain | 1 (Ref.) | 0.84 (0.58-1.23) | 1.09 (0.65-1.81) |  | 0.90 (0.57-1.43) | 1.01 (0.72-1.43) | 0.82 (0.55-1.21) |
| Thyroid | 1 (Ref.) | 0.81 (0.58-1.40) | 1.35 (0.71-2.57) |  | 0.99 (0.52-1.90) | 1.49 (0.93-2.38) | 1.36 (0.82-2.26) |
| Non-Hodgkin lymphoma | 1 (Ref.) | 0.94 (0.74-1.19) | 1.04 (0.75-1.44) |  | 0.89 (0.67-1.18) | 1.00 (0.80-1.24) | 0.93 (0.73-1.18) |
| Multiple myeloma | 1 (Ref.) | 0.74 (0.50-1.08) | 1.37 (0.87-2.16) |  | 0.60 (0.36-1.00) | 0.99 (0.71-1.39) | 1.05 (0.73-1.52) |
| Leukemia | 1 (Ref.) | 0.99 (0.72-1.35) | 0.72 (0.43-1.20) |  | 0.92 (0.63-1.34) | 1.22 (0.92-1.61) | 1.03 (0.76-1.42) |

Multivariable models were adjusted for sex, age, ethnicity, Townsend deprivation index, qualification, employment status, alcohol intaking, smoking status. Models for cervix, ovary and endometrium cancers are additionally adjusted for HRT use, oral contraceptive use and menopause after excluded females with history of hysterectomy. Models for postmenopausal breast was additionally adjusted for HRT and oral contraceptive use.

**Table 8: Multivariable HRs and 95% CIs for cancer** **across BMI and metabolic status where subjects who had a history of cardiovascular disease (heart attack, angina, and stroke) and diabetes at baseline were excluded.**

| Cancer type/sites | Metabolically healthy | | |  | Metabolically unhealthy | | |
| --- | --- | --- | --- | --- | --- | --- | --- |
|  | Normal weight | Overweight | Obesity |  | Normal weight | Overweight | Obesity |
| Oral | 1 (Ref.) | 0.89 (0.58-1.36) | 0.79 (0.41-1.54) |  | 1.30 (0.81-2.08) | 0.88 (0.58-1.33) | 1.26 (0.83-1.92) |
| Esophagus | 1 (Ref.) | 1.28 (0.86-1.89) | **2.14 (1.35-3.41)** |  | 1.21 (0.76-1.92) | **1.57 (1.10-2.24)** | **1.76 (1.21-2.56)** |
| Stomach | 1 (Ref.) | 1.30 (0.86-1.99) | 0.72 (0.35-1.50) |  | 0.97 (0.56-1.68) | 1.19 (0.79-1.78) | 1.42 (0.92-2.18) |
| Colorectal | 1 (Ref.) | 1.11 (0.97-1.26) | 1.11 (0.92-1.34) |  | 1.16 (0.99-1.35) | **1.18 (1.04-1.34)** | **1.24 (1.08-1.42)** |
| Liver | 1 (Ref.) | 0.88 (0.53-1.46) | 1.16 (0.59-2.25) |  | 1.54 (0.91-2.58) | 0.95 (0.59-1.52) | 1.50 (0.94-2.40) |
| Biliary tract | 1 (Ref.) | 1.52 (0.68-3.40) | 1.87 (0.67-5.21) |  | 0.55 (0.15-2.01) | 0.59 (0.23-1.51) | 1.95 (0.88-4.35) |
| Gallbladder | 1 (Ref.) | 1.41 (0.58-3.41) | 0.72 (0.15-3.35) |  | 0.66 (0.18-2.44) | 1.09 (0.44-2.68) | 1.34 (0.53-3.38) |
| Pancreas | 1 (Ref.) | 1.31 (0.96-1.78) | **1.60 (1.06-2.41)** |  | 1.05 (0.71-1.54) | 1.21 (0.90-1.64) | **1.64 (1.20-2.25)** |
| Lung | 1 (Ref.) | 0.89 (0.75-1.06) | 0.95 (0.74-1.21) |  | 1.16 (0.96-1.40) | 0.98 (0.84-1.15) | 0.85 (0.71-1.02) |
| Malignant melanoma | 1 (Ref.) | 1.18 (1.00-1.39) | 0.85 (0.64-1.12) |  | 0.85 (0.67-1.08) | 1.14 (0.96-1.35) | 1.00 (0.82-1.22) |
| Postmenopausal Breast | 1 (Ref.) | **1.15 (1.02-1.30)** | **1.35 (1.15-1.59)** |  | 1.07 (0.93-1.25) | **1.22 (1.08-1.37)** | **1.29 (1.14-1.47)** |
| Cervix | 1 (Ref.) | 1.43 (0.63-5.25) | 1.04 (0.29-3.70) |  | 2.16 (0.84-5.58) | 1.66 (0.68-4.04) | 2.08 (0.87-4.96) |
| Endometrium | 1 (Ref.) | **1.38 (1.06-1.81)** | **2.84 (2.11-3.82)** |  | 1.15 (0.80-1.63) | **1.70 (1.30-2.24)** | **3.87 (3.03-4.93)** |
| Ovary | 1 (Ref.) | 1.19 (0.89-1.58) | 1.18 (0.79-1.77) |  | 1.21 (0.84-1.75) | 1.05 (0.76-1.45) | 1.24 (0.89-1.72) |
| Prostate | 1 (Ref.) | 0.95 (0.86-1.05) | 0.91 (0.78-1.06) |  | 0.93 (0.83-1.05) | 0.99 (0.90-1.08) | 0.85 (0.77-0.95) |
| Kidney | 1 (Ref.) | 1.17 (0.87-1.58) | **1.65 (1.13-2.41)** |  | 1.17 (0.82-1.67) | **1.41 (1.07-1.86)** | **1.88 (1.41-2.50)** |
| Bladder | 1 (Ref.) | 1.15 (0.80-1.34) | 1.27 (0.78-2.07) |  | 1.38 (0.93-2.06) | 1.35 (0.98-1.86) | **1.54 (1.09-2.18)** |
| Brain | 1 (Ref.) | 0.78 (0.56-1.10) | 0.96 (0.61-1.54) |  | 0.98 (0.66-1.46) | 1.05 (0.77-1.42) | 0.63 (0.43-0.94) |
| Thyroid | 1 (Ref.) | 0.86 (0.53-1.38) | 1.64 (0.95-2.84) |  | 1.00 (0.54-1.84) | **1.92 (1.27-2.91)** | **1.41 (0.87-2.26)** |
| Non-Hodgkin lymphoma | 1 (Ref.) | 0.92 (0.75-1.14) | 1.01 (0.74-1.36) |  | 1.01 (0.78-1.31) | 1.01 (0.82-1.23) | 0.94 (0.75-1.18) |
| Multiple myeloma | 1 (Ref.) | 0.76 (0.54-1.08) | 1.39 (0.91-2.12) |  | 0.59 (0.37-0.96) | 0.83 (0.60-1.15) | 1.15 (0.82-1.61) |
| Leukemia | 1 (Ref.) | 0.88 (0.66-1.18) | 0.71 (0.45-1.13) |  | 0.95 (0.67-1.34) | 1.19 (0.92-1.54) | 1.06 (0.79-1.42) |

Multivariable models were adjusted for sex, age, ethnicity, Townsend deprivation index, qualification, employment status, alcohol intaking, smoking status. Models for cervix, ovary and endometrium cancers are additionally adjusted for HRT use, oral contraceptive use and menopause after excluded females with history of hysterectomy. Models for postmenopausal breast was additionally adjusted for HRT and oral contraceptive use.

**Table 9: Multivariable HRs and 95% CIs for cancer across BMI where missing values are imputed by multiple imputation.**

| Cancer type/sites | Normal weight | Overweight | Obesity |
| --- | --- | --- | --- |
| Endometrium | 1 (Ref.) | **1.56 (1.28-1.92)** | **3.53 (2.91-4.28)** |
| Biliary tract | 1 (Ref.) | 1.00 (0.52-1.87) | **2.12 (1.14-3.93)** |
| Kidney | 1 (Ref.) | **1.34 (1.09-1.64)** | **1.98 (1.60-2.45)** |
| Pancreas | 1 (Ref.) | 1.22 (0.99-1.51) | **1.58 (1.23-1.99)** |
| Esophagus | 1 (Ref.) | 1.26 (0.99-1.62) | **1.48 (1.12-1.93)** |
| Liver | 1 (Ref.) | 0.93 (0.68-1.27) | **1.44 (1.06-1.99)** |
| Bladder | 1 (Ref.) | 1.13 (0.88-1.40) | **1.45 (1.13-1.83)** |
| Stomach | 1 (Ref.) | 1.28 (0.95-1.72) | 1.34 (0.96-1.86) |
| Multiple myeloma | 1 (Ref.) | 0.99 (0.76-1.26) | **1.33 (1.02-1.76)** |
| Gallbladder | 1 (Ref.) | 1.21 (0.63-2.30) | 1.29 (0.62-2.60) |
| Postmenopausal Breast | 1 (Ref.) | **1.16 (1.08-1.23)** | **1.29 (1.18-1.39)** |
| Cervix | 1 (Ref.) | 1.09 (0.59-2.01) | 1.25 (0.63-2.44) |
| Thyroid | 1 (Ref.) | 1.22 (0.89-1.68) | 1.25 (0.87-1.81) |
| Colorectal | 1 (Ref.) | **1.11 (1.02-1.24)** | **1.20 (1.07-1.33)** |
| Ovary | 1 (Ref.) | 1.09 (0.88-1.34) | 1.16 (0.90-1.47) |
| Oral | 1 (Ref.) | 0.84 (0.62-1.12) | 1.03 (0.75-1.42) |
| Malignant melanoma | 1 (Ref.) | **1.17 (1.03-1.34)** | 1.04 (0.87-1.20) |
| Non-Hodgkin lymphoma | 1 (Ref.) | 0.99 (0.85-1.15) | 1.02 (0.86-1.21) |
| Leukemia | 1 (Ref.) | 1.08 (0.89-1.32) | 0.99 (0.78-1.25) |
| Lung | 1 (Ref.) | **0.88 (0.79-0.99)** | **0.86 (0.74-0.97)** |
| Prostate | 1 (Ref.) | 0.99 (0.94-1.06) | **0.85 (0.77-0.92)** |
| Brain | 1 (Ref.) | 0.95 (0.75-1.20) | 0.83 (0.63-1.11) |

Multivariable models were adjusted for sex, age, ethnicity, Townsend deprivation index, qualification, employment status, alcohol intaking, smoking status. Models for cervix, ovary and endometrium cancers are additionally adjusted for HRT use, oral contraceptive use and menopause after excluded females with history of hysterectomy. Models for postmenopausal breast was additionally adjusted for HRT and oral contraceptive use.

**Table 10: Multivariable hazard ratios and 95% confidence intervals for cancer across BMI and metabolic status where missing values are imputed by multiple imputation.**

| Cancer type/sites | | Metabolically healthy | | | | | | Metabolically unhealthy | | | | | |
| --- | --- | --- | --- | --- | --- | --- | --- | --- | --- | --- | --- | --- | --- |
|  | Normal weight | | Overweight | | Obesity | | Normal weight | | Overweight | | Obesity | |  |
| Oral | | 1 (Ref.) | | 0.85 (0.56-1.30) | | 0.91 (0.49-1.68) | | 1.28 (0.82-1.99) | | 0.94 (0.64-1.38) | | 1.18 (0.79-1.76) | |
| Esophagus | | 1 (Ref.) | | 1.19 (0.81-1.75) | | **2.01 (1.29-3.15)** | | 1.35 (0.89-2.03) | | **1.56 (1.12-2.18)** | | **1.50 (1.05-2.14)** | |
| Stomach | | 1 (Ref.) | | 1.35 (0.89-2.06) | | 0.80 (0.40-1.61) | | 1.05 (0.64-1.74) | | 1.31 (0.89-1.93) | | 1.47 (0.98-2.21) | |
| Colorectal | | 1 (Ref.) | | 1.12 (0.98-1.27) | | 1.11 (0.92-1.34) | | 1.12 (0.96-1.30) | | **1.18 (1.04-1.33)** | | **1.28 (1.12-1.45)** | |
| Liver | | 1 (Ref.) | | 0.94 (0.58-1.53) | | 1.26 (0.68-2.30) | | 1.31 (0.80-1.24) | | 1.01 (0.66-1.56) | | 1.29 (0.83-1.99) | |
| Biliary tract | | 1 (Ref.) | | 1.54 (0.76-3.12) | | 1.86 (0.72-4.78) | | 1.06 (0.93-1.20) | | 0.67 (0.31-1.45) | | **2.17 (1.14-4.12)** | |
| Gallbladder | | 1 (Ref.) | | 1.21 (0.51-2.86) | | 0.59 (0.13-2.70) | | 0.73 (0.24-2.16) | | 0.89 (0.39-2.07) | | 1.04 (0.44-2.46) | |
| Pancreas | | 1 (Ref.) | | 1.36 (1.00-1.84) | | **1.61 (1.08-2.42)** | | 1.22 (0.86-1.74) | | 1.28 (0.95-1.70) | | **1.65 (1.23-2.23)** | |
| Lung | | 1 (Ref.) | | 0.88 (0.74-1.04) | | 0.98 (0.78-1.24) | | 1.15 (0.97-1.37) | | 0.96 (0.83-1.12) | | **0.85 (0.72-1.00)** | |
| Malignant melanoma | | 1 (Ref.) | | 1.16 (0.98-1.37) | | 0.85 (0.65-1.11) | | 0.86 (0.68-1.07) | | 1.07 (0.90-1.26) | | 1.02 (0.85-1.23) | |
| Postmenopausal Breast | | 1 (Ref.) | | **1.10 (1.01-1.19)** | | **1.25 (1.11-1.40)** | | 1.01 (0.90-1.13) | | **1.16 (1.06-1.27)** | | **1.22 (1.11-1.34)** | |
| Cervix | | 1 (Ref.) | | 1.04 (0.50-2.17) | | 0.71 (0.21-2.40) | | 1.04 (0.85-1.25) | | 1.16 (0.54-2.51) | | 1.53 (0.74-3.14) | |
| Endometrium | | 1 (Ref.) | | **1.35 (1.04-1.76)** | | **2.52 (1.88-3.37)** | | 1.03 (0.73-1.45) | | **1.57 (1.21-2.03)** | | **3.16 (2.49-4.01)** | |
| Ovary | | 1 (Ref.) | | 1.13 (0.87-1.48) | | 1.16 (0.80-1.68) | | 1.03 (0.74-1.45) | | 1.05 (0.79-1.39) | | 1.16 (0.86-1.55) | |
| Prostate | | 1 (Ref.) | | 0.95 (0.86-1.04) | | 0.89 (0.76-1.03) | | 0.94 (0.84-1.05) | | 0.98 (0.90-1.07) | | **0.84 (0.76-0.93)** | |
| Kidney | | 1 (Ref.) | | 1.22 (0.91-1.64) | | **1.65 (1.14-2.39)** | | 1.08 (0.77-1.53) | | **1.42 (1.09-1.86)** | | **1.95 (1.49-2.56)** | |
| Bladder | | 1 (Ref.) | | 1.19 (0.85-1.68) | | 1.30 (0.81-2.08) | | 1.30 (0.90-1.89) | | 1.30 (0.96-1.77) | | **1.64 (1.19-2.26)** | |
| Brain | | 1 (Ref.) | | 0.79 (0.57-1.10) | | 1.00 (0.64-1.57) | | 0.92 (0.62-1.35) | | 0.99 (0.74-1.34) | | 0.74 (0.52-1.04) | |
| Thyroid | | 1 (Ref.) | | 0.80 (0.50-1.29) | | 1.50 (0.87-2.58) | | 1.21 (0.71-2.07) | | **1.76 (1.18-2.63)** | | 1.22 (0.77-1.94) | |
| Non-Hodgkin lymphoma | | 1 (Ref.) | | 0.92 (0.74-1.13) | | 0.96 (0.71-1.29) | | 0.97 (0.76-1.23) | | 0.99 (0.82-1.20) | | 0.98 (0.80-1.22) | |
| Multiple myeloma | | 1 (Ref.) | | 0.74 (0.52-1.04) | | 1.36 (0.90-2.04) | | **0.57 (0.36-0.89)** | | 0.85 (0.62-1.16) | | 1.00 (0.72-1.38) | |
| Leukemia | | 1 (Ref.) | | 0.88 (0.67-1.17) | | 0.74 (0.48-1.15) | | 0.91 (0.66-1.27) | | 1.15 (0.90-1.48) | | 1.00 (0.76-1.32) | |

Multivariable models were adjusted for sex, age, ethnicity, Townsend deprivation index, qualification, employment status, alcohol intaking, smoking status. Models for cervix, ovary and endometrium cancers are additionally adjusted for HRT use, oral contraceptive use and menopause after excluded females with history of hysterectomy. Models for postmenopausal breast was additionally adjusted for HRT and oral contraceptive use.

**Figure 1: Participant flow diagram.**

502 528

Total Participants

**Excluded participants with:**

| Prevalent cancer | 41491 |
| --- | --- |
| Body mass index < 18.5 kg/m^2^ | 2357 |

458 680

Total Participants

**Excluded missing values of exposures**

| **Variable** | **Missing** | **%** |
| --- | --- | --- |
| Body mass index | 2873 | 0.6 |
| Triglycerides | 28765 | 6.3 |
| HDL cholesterol | 36046 | 7.8 |
| Waist circumference | 64 | 0.0 |
| Glucose | 357 | 0.0 |

390 575

Total Participants included
